# Efficacy and safety of topical resorcinol 15% versus topical clindamycin 1% in the management of mild‐to‐moderate hidradenitis suppurativa: A retrospective study

**DOI:** 10.1111/dth.15439

**Published:** 2022-03-18

**Authors:** Elisa Molinelli, Valerio Brisigotti, Oriana Simonetti, Claudia Sapigni, Giovanni Marco D'Agostino, Giulio Rizzetto, Alfredo Giacchetti, Annamaria Offidani

**Affiliations:** ^1^ Dermatological Unit, Department of Clinical and Molecular Sciences Polytechnic Marche University Ancona Italy; ^2^ Dermatological Unit Institute for Treatment and Research (INRCA IRCCS) Ancona Italy

**Keywords:** antibiotic, antibiotic resistance, clindamycin, hidradenitis suppurativa, HS treatment, resorcinol

## Abstract

Topical and systemic antibiotic therapy remains the first‐line treatment for mild‐to‐moderate hidradenitis suppurativa (HS). However, literature data on antibiotic resistance in HS are growing. A total of 134 patients with mild‐to‐moderate HS were retrospectively evaluated. Seventy‐three patients (group A) received topical clindamycin 1% and 61 patients (group B) received topical resorcinol 15%. We evaluated the efficacy and tolerability of topical 15% resorcinol versus topical 1% clindamycin in mild‐to‐moderate HS, comparing the clinical response at 12 weeks of treatment. Patients treated with resorcinol 15% showed a significant improvement in Hidradenitis Suppurativa Clinical Response, International Hidradenitis Suppurativa Severity Score System, and Pain Visual Analogue Scale score from baseline compared to patients treated with clindamycin 1%. Topical resorcinol 15% could be a valid alternative to clindamycin in the management of acute and long‐standing HS, limiting antibiotic use and antimicrobial resistance.

## 1 INTRODUCTION

Hidradenitis suppurativa (HS) is a chronic inflammatory cutaneous disease involving the follicular areas of apocrine regions and clinically manifesting with nodules, abscesses, draining fistulas and scarring.^1,2^

Both topical and systemic antibiotics are still considered as first‐line therapy due to their anti‐inflammatory and immunomodulating activity, although this disease is not directly caused by bacterial infections. Topical clindamycin is suggested in the treatment of mild‐to‐moderate HS (Hurley stage I and stage II), although the evidences for this recommendation remains limited.^3^, ^4^, ^5^

The emergence of antimicrobial resistance, which can reduce the efficacy of antibiotics and increase vulnerability to infections, is also becoming a growing problem in HS patients, due to the massive use of antibiotics in the management of the disease.^6^, ^7^ In this scenario, alternative therapies should be introduced in the range of therapeutic possibilities of HS. Resorcinol has recently been described as a valid therapy for HS both in acute flares and long‐term disease.^8^

The aim of our study was to compare the efficacy and safety in long‐term use of 15% topical resorcinol versus 1% topical clindamycin in mild‐to‐moderate HS.

## 2 REPORT

Totally, 134 patients with mild‐to‐moderate HS (Hurley stage I and II) referred to our institution between January 2018 and December 2020 were retrospectively evaluated. Inclusion criteria required treatment with topical clindamycin 1% (gel or cream) or resorcinol 15% cream in monotherapy for at least 12 weeks and clinical follow‐up for longer than 3 months. Demographics, disease severity, and clinic response were recorded in a database, as well as data on adverse events and disease‐free survival. Previous treatment included topical antibiotics, systemic antibiotics (tetracycline, macrolides, and clindamycin plus rifampicin), isotretinoin, oral zinc and metformin. A total of 73 patients (group A) were treated with topical clindamycin 1% (gel) twice daily, while 61 patients (group B) received topical resorcinol 15% (galenic formulation, oil/water cream) once daily for 12 weeks applied on inflammatory nodules, abscesses, and tunnels (Table 1).

**TABLE 1:** Demographic and clinical characteristics of HS patients (group A: clindamycin 1% and group B: resorcinol 15%)

Factors		Group A	Group B
		*n* (%)	*n* (%)
Sex	Female	39 (53.4)	27 (44.3)
	Male	34 (46.6)	34 (55.7)
Average age (mean ± SD)		29.4 ± 7.7	32.2 ± 11.2
Average BMI (mean ± SD)		21.3 ± 3.7	29.2 ± 6.8
Smokers (%)		36 (49.3)	31 (50.8)
Age of onset (mean ± SD)		16.4 ± 4.1	18.7 ± 5.5
Disease duration, years (mean ± SD)		10.5 ± 6.7	13.1 ± 8.5
Family history (%)		30 (41.1)	21 (34.4)
Comorbidities	Acne	22 (30.1)	17 (27.9)
	Psoriasis	1 (1.4)	1 (1.6)
	Obesity	14 (19.2)	8 (13.1)
	Overweight	16 (21.9)	17 (27.9)
	Diabetes II	2 (2.7)	0
	Pilonidal cyst	14 (19.7)	12 (19.7)
	Hashimoto's disease	1 (1.4)	1 (1.6)
	PCOS	3 (4.1)	5 (8.2)
	Neurological/psychiatric disorders (epilepsy, schizophrenia)	3 (4.1)	1 (1.6)
	Arterial hypertension	2 (2.7)	1 (1.6)
	Systemic lupus erythematosus	0	1 (1.6)
	Rheumatoid arthritis	2 (2.7)	1 (1.6)
	Psoriatic arthritis	1 (1.4)	0
	IBD	2 (2.7)	3 (4.9)
No comorbidities (%)		31 (42.5)	26 (45.9)
Previous treatment	No previous treatment	39	29
	Topical antibiotics	11	9
	Tetracyclines	12	13
	Macrolides	2	1
	Oral zinc	0	2
	Isotretinoin	7	4
	Clindamycin plus rifampicin	2	2
	Metformin	0	1
Hurley stage (%)	I	34 (46.6)	26 (42.6)
	II	39 (53.4)	35 (57.4)
IHS4 (%)	Mild	36 (49.3)	29 (47.5)
	Moderate	37 (50.6)	32 (52.5)
Average IHS4 (mean ± SD)		3.7 ± 1.5	3.9 ± 1.4
HS‐SOS (%)	I	30 (41.1)	19 (31.1)
	II	43 (58.9)	42 (68.9)
Affected areas (%)	Axilla	23 (31.5)	16 (26.2)
	Groin	32 (43.8)	25 (41.0)
	Breast	4 (5.5)	8 (13.1)
	Buttocks	12 (16.4)	12 (19.7)
	Occipitocervical	2 (2.7)	1 (1.6)
	Abdomen	2 (2.7)	2 (3.3)

Abbreviations: BMI, body mass index; HS, hidradenitis suppurativa; HS‐SOS, Sonographic Scoring of Hidradenitis Suppurativa; IBD, inflammatory bowel disease; IHS4, International Hidradenitis Suppurativa Severity Score System; PCOS, polycystic ovary syndrome; SD, standard deviation.

The clinical response was evaluated through Hidradenitis Suppurativa Clinical Response (HiSCR) (as at least a 50% reduction from baseline in the total abscess and inflammatory nodule count, with no increase in the abscess or draining sinus tract count), Pain Visual Analogue Scale (VAS), Dermatology Life Quality Index (DLQI), and International Hidradenitis Suppurativa Severity Score System (IHS4) scores after 12 weeks.

In group A (clindamycin 1%), clinical response (HiSCR) was obtained in 38 (52%) of 73 patients after 12 weeks (*p* < 0.01). In group B (resorcinol 15%), clinical response was achieved in 52 (85.3%) of 61 patients after 12 weeks (*p* < 0.001). At 12 weeks, the clinical response to resorcinol 15% was higher than the response to topical antibiotic, with statistically significance (*p* < 0.001). Pain VAS score and IHS4 showed a statistically higher decrease after resorcinol 15% cream application (group B) compared to clindamycin treatment (group A) (*p* < 0.001). The disease‐free survival was significantly higher in group B than group A (Table 2).

**TABLE 2:** Patients' disease score at baseline and at week 12 and HiSCR achievement (group A: clindamycin 1% and group B: resorcinol 15%)

	Group A		Group B	
Score	T0 *n* (%)	T12 *n* (%)	T0 *n* (%)	T12 *n* (%)
IHS4, mean ± SD	3.7 ± 1.5	3.5 ± 1.1	3.9 ± 1.4	3.3 ± 2.8*
PAIN VAS, mean ± SD	7.0 ± 1.9	5.1 ± 1.3*	6.7 ± 1.8	0.4 ± 0.7***
DLQI, mean ± SD	17.2 ± 2.4	11.0 ± 2.9**	16.8 ± 4.8	1.5 ± 2.1***
Patients who achieved HiSCR				
Hurley stage I *n* (%)		18 (52.9)**		22 (84.6)***
Hurley stage II *n* (%)		20 (51.3)*		30 (85.7)***
Patients who achieved HiSCR				
IHS4 category mild *n* (%)		22 (61.1)**		24 (82.8)**
IHS4 category moderate *n* (%)		16 (43.2)*		25 (78.1)***
Number of patients *n* (%)		38 (52.0)*		52 (85.3)***
Average disease‐free survival, wk ± SD	8.3 ± 3.2		12.5 ± 7.2	

Abbreviations: DLQI, Dermatology Life Quality Index; HiSCR, Hidradenitis Suppurativa Clinical Response; IHS4, International Hidradenitis Suppurativa Severity Score System; SD, standard deviation; VAS, Visual Analogue Scale.

* *p* < 0.05.

** *p* < 0.01.

*** *p* < 0.001.

In group A, 11 patients (15%) reported mild irritation. In group B, mild‐to‐moderate irritation, desquamation, and brown pigmentation were reported by 21 (43%), 35 (57%), and 25 (41%) patients, respectively. However, none of the 134 patients interrupted the therapy.

## 3 DISCUSSION

Topical clindamycin 1% applied twice daily for 12 weeks is still considered one of the first line treatment of mild‐to‐moderate HS. It has activity against anaerobic, staphylococcal and streptococcal species, and it also reduce skin inflammation and prevent biofilm formation.^9^, ^10^ It has usefulness predominantly in the management of less severe lesions (nodules, follicular papules/pustules), rather than deep abscesses.^3^, ^9^

The emergence of bacterial resistance in HS is widely described in literature.^6^, ^7^, ^11^ Fisher et al.^6^ in a cross‐sectional analysis conducted on 239 patients reported a large proportion of patients using topical clindamycin developed resistant strains of *Staphylococcus aureus* compared with patients using no antibiotics. Hessam et al.^11^ retrospectively investigated bacterial cultures with antibiograms of samples obtained from deep portions of HS lesions, documenting that clindamycin and tetracycline were often associated with a higher rate of resistance (55% and 32.6% of bacterial cultures, respectively). More recently, Bettoli et al.^12^ demonstrated a high levels of resistance to rifampicin, clindamycin, and tetracyclines, supporting the fact that their beneficial effect is mainly due to the immunomodulatory or antimicrobial activity against the anaerobic bacteria that are prevalent in HS lesions. The authors concluded appropriately that a targeted and specific antibiotic therapy, driven by microbiological evaluations, seems more appropriate than empiric, generic, non‐specific, and therapeutic approaches.

The use of resorcinol (1,3‐dihydroxybenzene) which has antimicrobic, anti‐inflammatory, and keratolytic activities, was first described by Unna in 1882 as chemical peeling in the management of acne.^13^ Literature data on the efficacy of resorcinol in HS are increasing and it is mentioned both in the short and long‐term treatment of mild‐to‐moderate disease.^14^, ^15^

Topical resorcinol 15% has been suggested by Boer et al.^14^ as an effective self‐administered treatment in HS, with a significant reduction of pain and duration of disease flares. In a small perspective study using clinical and ultrasound evaluation, Pascual et al.^15^ showed the beneficial effect of resorcinol 15% applied two times daily for 30 days in acute HS manifestations (fistulous tracts were excluded).

Recently, we have revealed the efficacy of resorcinol 15% in short and long‐term management of non‐fistulous and fistulous HS manifestations.^8^ The current study corroborates the effectiveness of topical resorcinol as sustained treatment in HS manifestations compared to traditional clindamycin. Resorcinol may be a sensitizing agent.^8^ Our study also established that resorcinol in concentrations of 15% is a well‐tolerated therapeutic option; irritation, desquamation, and brown pigmentation were usually minimal. Systemic toxicity is very rare and it is associated with high concentrations (40%–50%) and when it is used in large amounts or on large injured surfaces.^14^, ^15^

Antibiotics should be administered only when clinically indispensable and they could be reserved for severe cases. Considering the serious risk of future increases in antibiotic resistance as well as dissatisfaction with clinical outcomes normally observed using topical antibiotics, resorcinol might represent an excellent alternative to topical clindamycin in flares and maintenance management of HS lesions. The main limitation of the study is its uncontrolled retrospective design. Randomized blinded studies are necessary to verify our results.

## CONFLICT OF INTEREST

The authors declare no potential conflict of interest.

## AUTHOR CONTRIBUTIONS

Annamaria Offidani and Alfredo Giacchetti conceived of the presented idea. Giovanni Marco D'Agostino wrote the manuscript with support from Giulio Rizzetto, Claudia Sapigni, and Valerio Brisigotti. Elisa Molinelli and Oriana Simonetti helped supervise the project. All authors discussed the results and contributed to the final manuscript.

## Data Availability

The data that support the findings of this study are available from the corresponding author, E.M., upon reasonable request.
